# Vitamin D Status and Survival in Stage II-III Colorectal Cancer

**DOI:** 10.3389/fonc.2020.581597

**Published:** 2020-12-17

**Authors:** Yichao Bao, Yaqi Li, Yan Gong, Qianxia Huang, Sanjun Cai, Junjie Peng

**Affiliations:** ^1^Department of Colorectal Surgery, Fudan University Shanghai Cancer Center, Shanghai, China; ^2^Department of Oncology, Shanghai Medical College, Fudan University, Shanghai, China; ^3^Department of Clinical Laboratory, Fudan University Shanghai Cancer Center, Shanghai, China

**Keywords:** colorectal cancer, 25-hydroxyvitamin D3[25(OH)D], survival, stage III, vitamin D status

## Abstract

Vitamin D status has been shown to be positively correlated with the morbidity and prognosis of colorectal cancer (CRC) patients. However, the prognostic effect of vitamin D status on patients with stage II and III CRC, especially Asian patients, remains unclear. A total of 728 patients (523 in the primary cohort and 205 in the validation cohort) who were diagnosed with stage II-III CRC between January 2011 and December 2015 were enrolled. Their serum 25-hydroxyvitamin D3 [25(OH)D] levels were tested. Kaplan-Meier curves and Cox regression analyses were carried out. Subgroup analyses were conducted according to tumor location. In the primary cohort, the serum 25(OH)D level was positively correlated with the overall survival (OS) of all CRC patients (p= 0.016) and stage III patients (p= 0.009), while no correlation was found between 25(OH)D level and the prognosis of patients with stage II CRC. Moreover, 25(OH)D level was an independent prognostic factor for the OS of all patients with CRC [HR 0.541, 95% CI 0.334–0.875, p=0.012] and those with stage III CRC (HR 0.563, 95% CI 0.319–0.993, p=0.047). Subgroup analysis indicated that only in the left-sided subgroup, stage III CRC patients with high 25(OH)D levels had better OS than those with low 25(OH)D levels (HR 0.474, 95% CI 0.230–0.978, p=0.043). In the validation cohort, serum 25(OH)D levels were verified to have prognostic value for patients with stage III CRC (HR 0.220, 95% CI 0.080–0.602, p=0.003), and low 25(OH)D levels indicated worse OS for left-sided stage III CRC patients (HR 0.233, 95% CI 0.075–0.727, p=.012). In conclusion, vitamin D status is positively correlated with the survival of CRC patients, especially those with left-sided stage III CRC.

## Introduction

Vitamin D is a fat-soluble steroid hormone precursor that can be obtained from food and is synthesized by the skin through sunlight and ultraviolet radiation. The main form of stored vitamin D in the human body is 25-hydroxyvitamin D3 [25(OH)D]. In the human body, vitamin D3 and vitamin D2 bind to vitamin D-binding protein in plasma and are transported to the liver. These proteins are hydroxylated into vitamin D (25-OH), namely, 25-hydroxyvitamin D (25 (OH) D), a metabolite that can be detected in the blood. As the main form of stored vitamin D in the body, serum 25(OH)D can be tested to determine total vitamin D status. An epidemiological survey showed that vitamin D nutritional status is unsatisfactory in Asian populations, indicating a high proportion of patients with vitamin D deficiency (1–5).

Colorectal cancer (CRC) is the third most common cancer and has the second highest mortality rate worldwide, imposing an increasingly heavy burden on patients (6). Increasing evidence indicates a positive correlation between vitamin D status in the human body and the morbidity of CRC (7, 8). A meta-analysis of 52 trials with a total of 75,454 participants showed through well-grounded statistics that vitamin D supplementation significantly reduced the risk of cancer mortality (HR 0.84, 95% CI 0.74–0.95) by 16% (9).

In a pilot retrospective study, higher 25(OH)D levels were associated with improved overall survival (OS) in CRC patients (10–12), and vitamin D deficiency was very common in patients with stage IV colorectal cancer who received first-line chemotherapy (13). However, vitamin D status has been less studied in Asian populations. The effect of vitamin D status on the survival and recurrence of patients with stage II and III CRC, especially Asian patients, is unknown. Therefore, stage II and III CRC patients from Fudan University Shanghai Cancer Center (FUSCC) were evaluated to study the relationship between serum 25(OH)D levels and patient prognosis, and subgroup analysis based on tumor location was performed.

## Methods

### Study Population

This study retrospectively enrolled two independent cohorts of consecutive patients during different time periods from FUSCC. The primary cohort comprised patients admitted from January 2011 to December 2013 to define a cut-off value of 25(OH)D and its prognostic value. The validation cohort comprised patients admitted from January 2014 to December 2015 to verify the cut-off value and to confirm the prognostic efficacy of 25(OH)D for stage III disease.

In both cohorts, the inclusion criteria were as follows: age between 18 and 80 years; pathologically confirmed colorectal adenocarcinoma, mucinous adenocarcinoma, or signet-ring cell carcinoma classified as stage II–III according to the 8th edition of the AJCC/UICC TNM staging system; and radical resection of the primary tumor. The exclusion criteria were also the same for both cohorts and were as follows: had emergency surgery because of an acute intestinal obstruction, bleeding or perforation; had evidence of distant metastases; received neoadjuvant therapy; had a history of other malignancies; and did not have available tissue specimens or follow-up data. This study was approved by the Institutional Review Board of FUSCC. All patients provided written informed consent. Patient demographic and clinicopathological variables were retrieved from the FUSCC database. In the primary cohort, we analyzed stage II and III patients, while only stage III patients were analyzed for subsequent research purposes.

The patients were followed up regularly according to CSCO guidelines for CRC. This study analyzed prognosis, overall survival (OS) and relapse-free survival (RFS). The survival data were provided by the Clinical Statistics Center of FUSCC, relying on the hospital medical records follow-up platform or contact with patients by phone or email. Patients who were alive at the last follow-up were censored for the analysis.

### Serum 25(OH)D Assessment

Serum was extracted from the latest blood samples collected from patients before surgery using standard sampling tubes. Electrochemiluminescence binding assays were carried out using **Elecsys** and **Cobase** (REF 05894913 190) immunoassay analyzers for 25(OH)D concentration measurements. All samples were tested in the FUSCC clinical laboratory. The patients in the primary and validation cohorts were tested separately and independently. Controls for the various concentration ranges were run individually according to the instructions.

### Statistical Analysis

Categorical variables were compared using the two-sided Pearson χ^2^ test or Fisher’s exact test as appropriate. The serum 25(OH)D level was analyzed as a continuous variable and compared using a *t* test or the Wilcoxon rank test as appropriate. Summary statistics on time-to-event variables were calculated according to the Kaplan-Meier method and compared by the log-rank test. Proportional hazards assumption is stated for valid estimates from Cox proportional hazards models in **Figure S2**. Cox regression was used for univariate and multivariate analyses with hazard ratios (HRs) and 95% confidence intervals (CIs). All analyses were performed using IBM SPSS Statistics software, version 23 (SPSS Inc., Chicago, IL, USA) and GraphPad Prism, version 8 (La Jolla, CA, USA). All P values were two-sided and considered significant when <0.05.

## Results

### Baseline Characteristics

**Figure 1:**
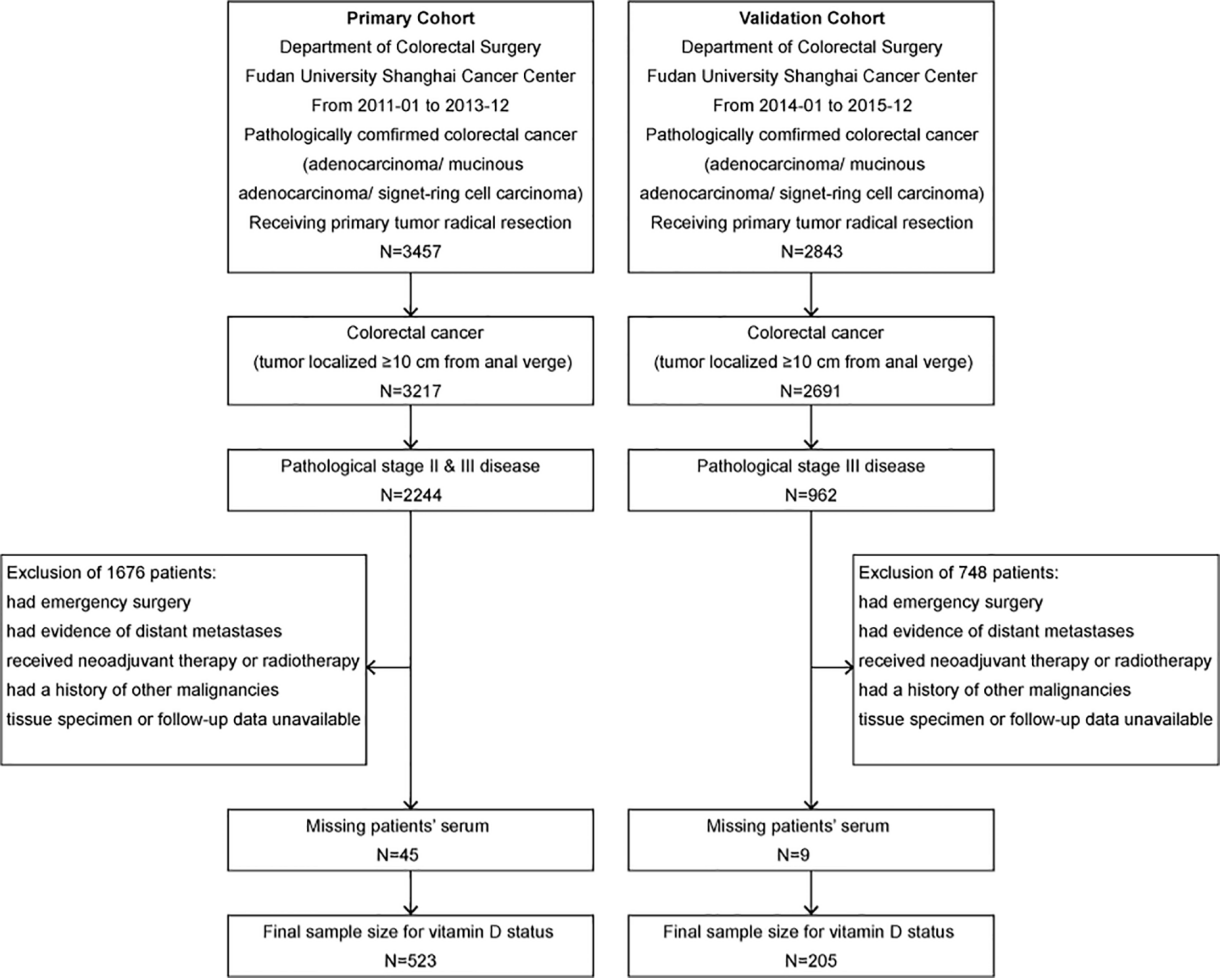
Flow diagram of the primary cohort and the validation cohort selection process. The inclusion and exclusion criteria have been described. The pathologic stage was redetermined according to the 8th edition of the AJCC/UICC TNM staging system.

In this study, the primary cohort consecutively enrolled 523 stage II–III CRC patients, including 244 (46.7%) stage II patients and 279 (53.3%) stage III patients, and the validation cohort consecutively enrolled 205 stage III CRC patients. The flow diagram of the cohort selection process is shown in **Figure 1**. The median age of the patients in the primary and validation cohorts was 61 years old (primary: range, 20–90, IQR, 52, 68; validation: range, 28–88, IQR, 52, 67). By December 2017, the median follow-up time in the primary cohort was 64.7 months (range, 4.4–102.8; IQR, 55.5, 79.2). By December 2019, the median follow-up time in the validation cohort was 53.0 months (range, 5.5–67.5; IQR, 46.3, 57.7). In the primary cohort, 74 (14.1%) patients suffered from recurrence (local recurrence or distant metastases), and 81 (15.5%) patients died, including 27 (5.2%) deaths without evidence of recurrence, while the 3-year OS rate was 91.7%, the 5-year OS rate was 85.7%, the 3-year RFS rate was 79.5%, and the 5-year RFS rate was 75.4%. In the validation cohort, 27 (12.6%) patients suffered from recurrence, and 48 (22.4%) patients died, including 11 (5.1%) without recurrence. The 3-year OS rate was 87.6%, the 5-year OS rate was 79.4%, the 3-year RFS rate was 73.8%, and the 5-year RFS rate was 70.8%. The baseline demographics and clinicopathologic characteristics of both cohorts are shown in **Table 1**.

**Table 1:** Baseline characteristics of patients with colorectal cancer (CRC) by serum 25(OH)D level.

Characteristic	Primary cohort			P	Validation cohort			P
	Cases	25(OH)D-Low	25(OH)D-High		Cases	25(OH)D-Low	25(OH)D-High	
	N (%)	N (%)	N (%)		N (%)	N (%)	N (%)	
No. of patients	523 (100.0)	290 (55.4)	233 (44.6)		205 (100.0)	125 (61.0)	80 (39.0)	
Age				0.543				0.967
<60	228 (43.6)	123 (53.9)	105 (46.1)		97 (47.3)	59 (60.8)	38 (39.2)	
≥60	295 (56.4)	167 (56.6)	128 (43.4)		108 (52.7)	66 (61.1)	42 (38.9)	
Sex				0.009				0.018
Male	304 (58.1)	154 (50.7)	150 (49.3)		120 (58.5)	65 (54.2)	55 (45.8)	
Female	219 (41.9)	136 (62.1)	83 (37.9)		85 (41.5)	60 (70.6)	25 (29.4)	
Histology				0.555				0.270
Adenocarcinoma	432 (82.6)	237 (54.9)	195 (45.1)		176 (85.9)	110 (62.5)	66 (37.5)	
Mucinous tumors	91 (17.4)	53 (58.2)	38 (41.8)		29 (14.1)	15 (51.7)	14 (48.3)	
Primary site				0.036				0.035
Left-sided	338 (64.6)	176 (52.1)	162 (47.9)		152 (74.1)	86 (56.6)	66 (43.4)	
Right-sided	185 (35.4)	114 (61.6)	71 (38.4)		52 (25.4)	38 (73.1)	14 (26.9)	
Stage				0.087				
II	244 (46.7)	145 (59.4)	99 (40.6)					
III	279 (53.3)	145 (52.0)	134 (48.0)					
T stage				0.980				0.320
Tis-T2	26 (5.0)	14 (53.8)	12 (46.2)		14 (6.8)	7 (50.0)	7 (50.0)	
T3	197 (37.7)	110 (55.8)	87 (44.2)		165 (80.5)	99 (60.0)	66 (40.0)	
T4	300 (57.4)	166 (55.3)	134 (44.7)		26 (12.7)	19 (73.1)	7 (26.9)	
N stage				0.272				0.530
N0	245 (46.8)	145 (59.2)	100 (40.8)					
N1	196 (37.5)	102 (52.0)	94 (48.0)		136 (66.3)	85 (62.5)	51 (37.5)	
N2	82 (15.7)	43 (52.4)	39 (47.6)		69 (33.7)	40 (58.0)	29 (42.0)	
Adjuvant chemotherapy				0.130				0.959
Yes	413 (79.0)	222 (53.8)	191 (46.2)		181 (88.3)	111 (61.3)	70 (38.7)	
No	110 (21.0)	68 (61.8)	42 (38.2)		21 (10.2)	13 (61.9)	8 (38.1)	
No. of LNs dissected				0.163				0.383
<12	44 (8.4)	20 (45.5)	24 (54.5)		14 (6.8)	7 (50.0)	7 (50.0)	
≥12	479 (91.6)	270 (56.4)	209 (43.6)		191 (93.2)	118 (61.8)	73 (38.2)	
Pathological grading				0.301				0.130
Well/moderate	382 (73.0)	207 (54.2)	175 (45.8)		150 (73.2)	97 (64.7)	53 (35.3)	
Poor/anaplastic	123 (23.5)	70 (56.9)	53 (43.1)		49 (23.9)	26 (53.1)	23 (46.9)	
Unknown	18 (3.4)	13 (72.2)	5 (27.8)		6 (2.9)	2 (33.3)	4 (66.7)	
Venous invasion				0.163				0.623
Negative	404 (77.2)	233 (57.7)	171 (42.3)		129 (62.9)	77 (59.7)	52 (40.3)	
Positive	112 (21.4)	54 (48.2)	58 (51.8)		76 (37.1)	48 (63.2)	28 (36.8)	
Unknown	7 (1.3)	3 (42.9)	4 (57.1)					
Perineural invasion				0.350				0.023
Negative	409 (78.2)	233 (57.0)	176 (43.0)		137 (66.8)	91 (66.4)	46 (33.6)	
Positive	111 (21.2)	55 (49.5)	56 (50.5)		68 (33.2)	34 (50.0)	34 (50.0)	
Unknown	3 (0.6)	2 (66.7)	1 (33.3)					
CEA (ng/ml)				0.615				0.463
≤6	316 (60.4)	178 (56.3)	138 (43.7)		78 (38.0)	47 (60.3)	31 (39.7)	
>5	203 (38.8)	109 (53.7)	94 (46.3)		99 (48.3)	58 (58.6)	41 (41.4)	
Unknown	4 (0.8)	3 (75.0)	1 (25.0)		28 (13.7)	20 (71.4)	8 (28.6)	
MMR status				0.102				0.698
pMMR	462 (88.3)	249 (53.9)	213 (46.1)		165 (80.5)	99 (60.0)	66 (40.0)	
dMMR	55 (10.5)	36 (65.5)	19 (34.5)		11 (5.4)	8 (72.7)	3 (27.3)	
Unknown	6 (1.1)	5 (83.3)	1 (16.7)		29 (14.1)	18 (62.1)	11 (37.9)	
Season				0.011				0.035
Spring	122 (23.3)	85 (69.7)	37 (30.3)		63 (30.7)	46 (73.0)	17 (27.0)	
Summer	169 (32.3)	95 (56.2)	74 (43.8)		54 (26.3)	30 (55.6)	24 (44.4)	
Fall	151 (28.9)	58 (38.4)	93 (61.6)		52 (25.4)	25 (48.1)	27 (51.9)	
Winter	81 (15.5)	52 (64.2)	29 (35.8)		36 (17.6)	24 (66.7)	12 (33.3)	

25(OH)D ranges are as follow: The median level was used to define the threshold value of the serum 25OHD level of the Chinese population.

25(OH)D, 25-hydroxyvitamin D3; LN, lymph node; CEA, Carcinoembryonic antigen; pMMR, proficient Mismatch Repair; dMMR, different Mismatch Repair.

### 25(OH)D Distribution

Since there is no consensus on the cut-off value for 25(OH)D according to references (10, 13–15), 25(OH)D ≥75 nmol/L was defined as an adequate level, ≥50 nmol/L and <75 nmol/L as a deficiency, and <50 nmol/L was defined as a lack of vitamin D. Using this standard, 58.5% of the study population had a shortage of vitamin D (<50 nmol/L), 30.4% showed a deficiency of vitamin D (<75 nmol/L), and only 11.1% had adequate vitamin D levels (≥75 nmol/L).

**Figure 2:**
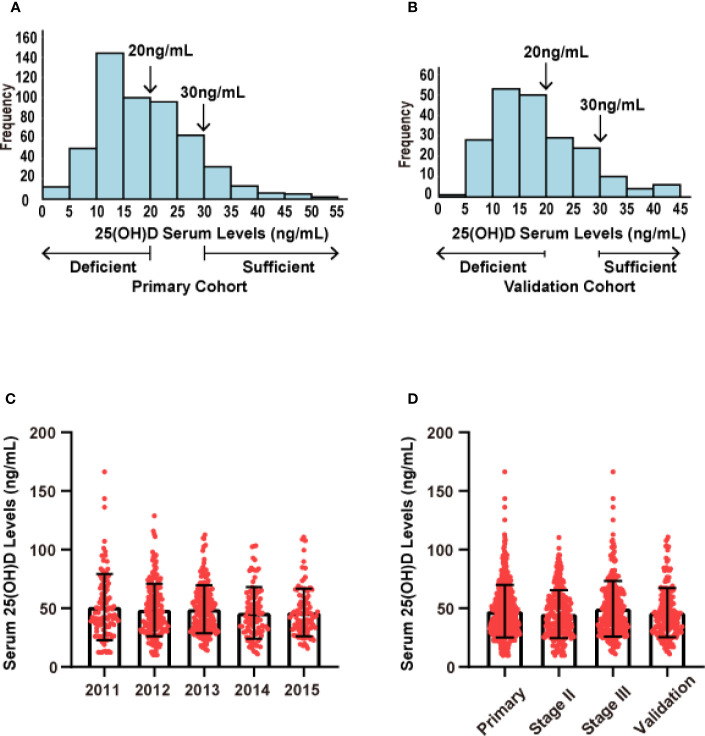
Distribution of serum 25(OH)D levels in both cohorts. **(A)** Distribution of serum 25(OH)D levels in the primary cohort (N = 523). **(B)** Distribution of serum 25(OH)D levels in the validation cohort (N = 205). **(C)** Distribution of serum 25(OH)D levels across different years of collected samples. **(D)** Distribution of serum 25(OH)D levels across different stages.

In our research on CRC prognosis, the median level of serum 25(OH)D in the primary cohort was used as the cut-off point. The median serum 25(OH)D was 47.50 nmol/L (range, 10.24 to 137.45 nmol/L). The distribution of serum 25(OH)D levels in both cohorts roughly conformed to the normal distribution (**Figure 2**). The patients’ demographic and clinicopathological variables are shown in **Table 1**. The serum 25(OH)D levels in female patients were significantly lower than those in male patients (median, 39.16 vs. 46.92 nmol/L, respectively; p = 0.009; **Table 1**). Patients with right-sided tumors showed significantly lower levels of 25(OH)D than those with left-sided tumors (median, 38.63 vs 46.20 nmol/L, respectively; p = 0.036; **Table 1**). According to previous studies (16), the level of 25(OH)D fluctuates with every season, among which winter and spring have relatively low levels, and autumn has the highest level (Primary cohort: p= 0.003; Validation cohort: p= 0.011) (**Supplementary Figure S1**), based on monthly comparisons of collected blood samples.

### Serum 25(OH)D as a Prognostic Biomarker for Stage III CRC

**Figure 3:**
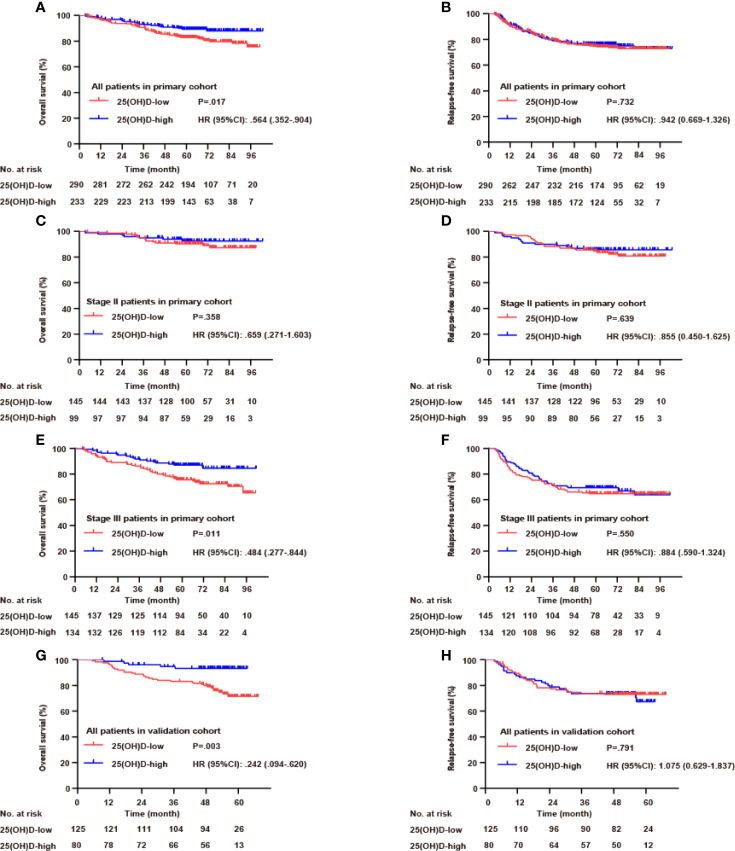
Serum 25(OH)D as a prognostic biomarker in both cohorts. Overall survival **(A)** and relapse-free survival **(B)** curves according to serum 25(OH)D levels among patients with CRC in the primary cohort. Overall survival **(C)** and relapse-free survival **(D)** curves according to serum 25(OH)D levels among patients with stage II CRC in the primary cohort. Overall survival **(E)** and relapse-free survival **(F)** curves according to serum 25(OH)D levels among patients with stage III CRC in the primary cohort. Overall survival **(G)** and relapse-free survival **(H)** curves according to serum 25(OH)D levels among patients with CRC in the validation cohort.

In the whole primary cohort, serum 25(OH)D level was a significant prognostic biomarker for OS (p=0.017, **Figure 3A**) but not for RFS (p=0.732, **Figure 3B**). For stage III disease, the serum 25(OH)D level was positively correlated with OS (p=0.011, **Figure 3E**) but not RFS (p=0.550, **Figure 3F**). However, for stage II disease, there were no significant associations with OS (p=0.358, **Figure 3C**) or RFS (p=0.639, **Figure 3D**). In the validation cohort, the prognostic value of serum 25(OH)D for stage III disease was verified. Serum 25(OH)D level was also a significant prognostic biomarker for OS (p=0.003, **Figure 3G**) but not for RFS (p=0.791, **Figure 3H**).

### Serum 25(OH)D as an Independent Prognostic Factor for OS in Stage III CRC

Next, Cox regression analysis was carried out for OS in both cohorts. In the whole primary cohort, univariate analysis showed that OS was associated with serum 25(OH)D level, age, histological type, N stage, number of lymph nodes examined and adjuvant chemotherapy (p<0.05). All these factors were included in the multivariate analysis, but only serum 25(OH)D level, age (HR 1.947, 95% CI 1.179–3.215, p=0.009), N stage (HR 2.261, 95% CI 1.659–3.081, p<0.001), number of lymph nodes examined (HR 2.537, 95% CI 1.367–4.707, p=0.003) and adjuvant chemotherapy (HR 0.485, 95% CI 0.285–0.825, p=0.008) were identified as independent factors (**Table 2**).

**Table 2:** Multivariate Cox regression analysis for overall survival of CRC in primary cohort.

Characteristic	Primary cohort			
	Univariate analysis	P value	Multivariate analysis	P value
	HR (95%CI)		HR (95%CI)	
Age		0.005		0.009
<60	1.000		1.000	
≥00	1.979 (1.228–3.191)		1.947 (1.179–3.215)	
Sex		0.605		0.936
Male	1.000		1.000	
Female	1.123 (0.724–1.740)		0.982 (0.629–1.533)	
Histology		0.030		0.096
Adenocarcinoma	1.000		1.000	
Mucinous tumors	1.736 (1.056–2.853)		1.543 (0.926–2.571)	
Primary site		0.383		
Right-sided	1.000			
Left-sided	0.820 (0.525-1.280)			
T stage		0.305		0.412
Tis-T2	1.000		1.000	
T3	1.119 (0.337–3.717)		1.836 (0.534–6.306)	
T4	1.587 (0.496–5.078)		2.133 (0.655–6.942)	
N stage		<0.001		<0.001
N0	1.000		1.000	
N1	1.874 (1.104–3.182)		2.413 (1.381–4.215)	
N2	3.592 (2.027–6.366)		4.911 (2.638–9.139)	
Adjuvant chemotherapy		0.042		0.008
No	1.000		1.000	
Yes	0.606 (0.374–0.983)		0.485 (0.285–0.825)	
No. of LNs dissected		0.005		0.003
≥03	1.000		1.000	
<12	2.326 (1.284–4.212)		2.537 (1.367–4.707)	
Pathological grading		0.095		
Well/moderate	1.000			
Poor/anaplastic	1.524 (0.939–2.472)			
Venous invasion		0.643		
Negative	1.000			
Positive	1.266 (0.763–2.100)			
Perineural invasion		0.601		
Negative	1.000			
Positive	0.957 (0.553–1.656)			
CEA (ng/ml)		0.340		
≤4	1.000			
>5	1.388 (0.896-2.150)			
MMR status		0.392		
pMMR	1.000			
dMMR	0.532 (0.215–1.315)			
Season		0.130		
Winter	1.000			
Spring	1.724 (0.935–3.176)			
Summer	1.520 (0.799–2.889)			
Fall	0.811 (0.344–1.913)			
25(OH)D level (ng/ml)		0.017		0.012
Low	1.000		1.000	
High	0.564 (0.352–0.904)		0.541 (0.334–0.875)	

+Calculated by using serum 25(OH)D as a continuous variable.

AJCC/UICC TNM staging system is highly related to T/N stage, there it is not included in multivariate analysis.

25(OH)D, 25-hydroxyvitamin D3; HR, hazard ratio; LN, lymph node; CEA, Carcinoembryonic antigen; pMMR, proficient Mismatch Repair; dMMR, different Mismatch Repair.

For stage III disease, univariate analysis showed that OS was associated with serum 25(OH)D level, age, histological type, primary site, N stage, adjuvant chemotherapy and perineural invasion (p<0.05). These factors were included in the multivariate analysis, but only serum 25(OH)D level (HR 0.563, 95% CI 0.319–0.993, p=0.047), age (HR 1.997, 95% CI 1.126–3.542, p=0.018), N stage (HR 1.900, 95% CI 1.098–3.286, p=0.022), adjuvant chemotherapy (HR 0.471, 95% CI 0.245–0.906, p=0.024) and perineural invasion (HR 0.795, 95% CI 0.424–1.493, p=0.043) were identified as independent factors (**Table 3**). Serum 25(OH)D levels were not prognostic factors for OS in stage II disease (HR 0.659, 95% CI 0.271–1.603, p=0.358) (**Table S1**).

**Table 3:** Multivariate Cox regression analysis for overall survival of stage III CRC in both cohorts.

Characteristic	Primary cohort				Validation cohort				
	Univariate analysis	P	Multivariate analysis	P	Univariate analysis	P	Multivariate analysis	P
	HR (95%CI)		HR (95%CI)		HR (95%CI)		HR (95%CI)	
Age		0.010		0.018		0.752		0.718
<60	1.000		1.000		1.000		1.000	
≥00	2.037 (1.183–3.508)		1.997 (1.126–3.542)		0.901 (0.473–1.718)		0.883 (0.447–1.741)	
Sex		0.532		0.922		0.806		0.734
Male	1.000		1.000		1.000		1.000	
Female	1.179 (0.704–1.973)		1.027 (0.598–1.767)		1.085 (0.566–2.079)		0.888 (0.448–1.759)	
Histology		0.018		0.240		0.287		0.155
Adenocarcinoma	1.000		1.000		1.000		1.000	
Mucinous tumors	2.099 (1.129–3.575)		1.448 (0.781–2.687)		1.564 (0.687–3.562)		1.910 (0.784–4.654)	
Primary site		0.049		0.284		0.035		0.529
Right-sided	1.000		1.000		1.000		1.000	
Left-sided	0.586 (0.345–0.997)		0.739 (0.425–1.285)		0.485 (0.248–0.949)		0.778 (0.355–1.701)	
T stage		0.510		0.508		<0.001		0.009
Tis-T2	1.000		1.000		1.000		1.000	
T3	2.000 (0.579–6.913)		2.093 (0.599–7.313)		0.634 (0.190–2.119)		0.599 (0.135–2.667)	
T4	1.985 (0.614–6.421)		1.924 (0.581–6.376)		2.955 (0.832–10.495)		2.122 (0.415–10.864)	
N stage		0.013		0.022		0.004		0.001
N1	1.000		1.000		1.000		1.000	
N2	1.933 (1.149–3.253)		1.900 (1.098–3.286)		2.594 (1.358–4.953)		4.064 (1.784–9.261)	
Adjuvant chemotherapy		0.004		0.024		0.005		0.007
No	1.000		1.000		1.000		1.000	
Yes	0.400 (0.216–0.742)		0.471 (0.245–0.906)		0.379 (0.193–0.747)		0.309 (0.133–0.720)	
No. of LNs dissected		0.051				0.541		
≥41	1.000				1.000			
<12	1.971 (0.996–3.899)				1.447 (0.443–4.724)			
Pathological grading		0.147				0.756		
Well/moderate	1.000				1.000			
Poor/anaplastic	1.561 (0.898–2.714)				1.322 (0.637–2.743)			
Venous invasion		0.968				0.039		0.915
Negative	1.000				1.000		1.000	
Positive	1.040 (0.599–1.805)				1.972 (1.035–3.759)		0.957 (0.427–2.148)	
Perineural invasion		0.006		0.043		0.365		
Negative	1.000		1.000		1.000			
Positive	0.824 (0.444–1.530)		0.795 (0.424–1.493)		0.707 (0.333–1.498)			
CEA (ng/ml)		0.769				0.012		0.072
≤7	1.000				1.000		1.000	
>5	1.210 (0.721–2.031)				1.172 (0.544–2.525)		1.107 (0.484–2.532)	
MMR status		0.123				0.632		
pMMR	1.000				1.000			
dMMR	0.210 (0.029–1.523)				0.447 (0.061–3.279)			
25(OH)D level (ng/ml)		0.011		0.047		0.003		0.003
Low	1.000		1.000		1.000		1.000	
High	0.484 (0.277–0.844)		0.563 (0.319–0.993)		0.242 (0.094–0.620)		0.220 (0.080–0.602)	

AJCC/UICC TNM staging system is highly related to T/N stage, there it is not included in multivariate analysis.

25(OH)D, 25-hydroxyvitamin D3; HR, hazard ratio; LN, lymph node; CEA, Carcinoembryonic antigen; pMMR, proficient Mismatch Repair; dMMR, different Mismatch Repair.

In the validation cohort, it was confirmed that serum 25(OH)D level was an independent factor for OS. Univariate analysis showed that OS was associated with serum 25(OH)D level, primary site, T stage, N stage, venous invasion, CEA level and adjuvant chemotherapy (p<0.05). These factors were included in the multivariate analysis, but only serum 25(OH)D level (HR 0.220, 95% CI 0.080–0.602, p=0.003), T stage (HR 2.252, 95% CI 1.059–4.792, p=0.009), N stage (HR 4.064, 95% CI 1.784–9.261, p=0.001), and adjuvant chemotherapy (HR 0.309, 95% CI 0.133–0.720, p=0.007) were identified as independent factors (**Table 3**).

### Subgroup Analysis of Serum 25(OH)D as a Prognostic Biomarker

**Figure 4:**
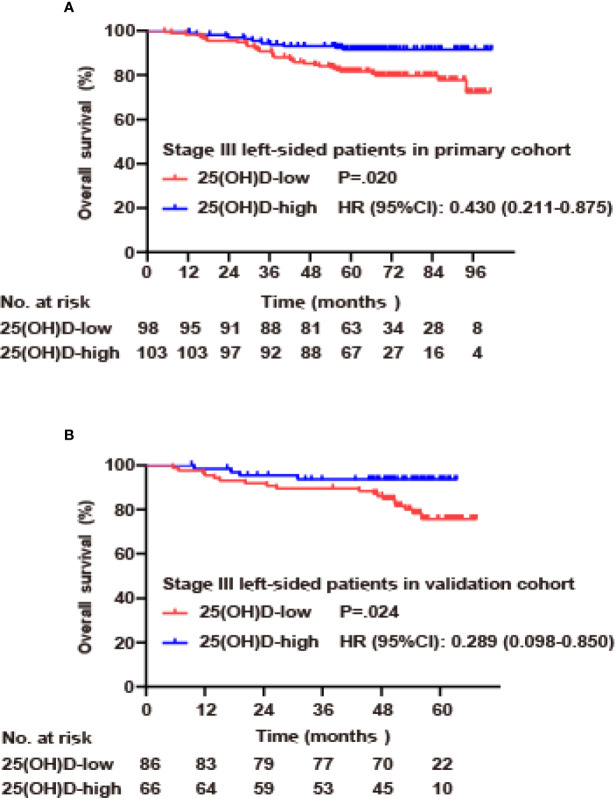
Serum 25(OH)D as an independent prognostic factor in stage III CRC. **(A)** Overall survival curves according to serum 25(OH)D levels among patients with left-sided stage III CRC in the primary cohort. **(B)** Overall survival curves according to serum 25(OH)D levels among patients with left-sided stage III CRC in the validation cohort.

Due to the large differences in biological characteristics and clinical characteristics between patients with right- and left-sided CRC (17–19), a subgroup analysis was carried out according to tumor location for stage III disease. In the primary cohort, for left-sided CRC, the serum 25(OH)D level was positively correlated with OS (p=0.020, **Figure 4A**). Using Cox regression analysis, univariate analysis showed that OS was associated with serum 25(OH)D level, age and adjuvant chemotherapy (p<0.05). These factors were included in the multivariate analysis, but only serum 25(OH)D level (HR 0.474, 95% CI 0.230–0.978, p=0.043) and adjuvant chemotherapy (HR 0.341, 95% CI 0.151–0.774, p=0.010) were identified as independent factors (**Figure 4A**, **Table 4**). For right-sided CRC, there was no significant association between serum 25(OH)D level and OS (p=0.392, **Table S2**).

**Table 4:** Univariate and multivariate Cox regression analysis for overall survival of CRC in stage III left-sided disease.

Characteristic	Primary cohort				Validation cohort				
	Univariate analysis	P	Multivariate analysis	P	Univariate analysis	P	Multivariate analysis	P
	HR (95%CI)		HR (95%CI)		HR (95%CI)		HR (95%CI)	
Age		0.017		0.061		0.798		0.627
<60	1.000		1.000		1.000		1.000	
≥00	2.335 (1.163–4.688)		2.015 (0.968–4.195)		0.898 (0.396–2.037)		0.809 (0.344–1.901)	
Sex		0.825		0.956		0.441		0.974
Male	1.000		1.000		1.000		1.000	
Female	1.078 (0.555–2.094)		1.019 (0.520–1.998)		1.383 (0.606–3.156)		0.986 (0.419–2.318)	
Histology		0.447		0.921		0.645		0.994
Adenocarcinoma	1.000		1.000		1.000		1.000	
Mucinous tumors	1.406 (0.584–3.384)		0.954 (0.375–2.424)		0.711 (0.167–3.032)		1.006 (0.216–4.675)	
T stage		0.135		0.249		0.007		0.007
Tis-T2	1.000		1.000		1.000		1.000	
T3	5.659 (0.744–43.051)		5.082 (0.649–39.802)		0.749 (0.170–3.300)		0.560 (0.122–2.579)	
T4	3.474 (0.466–25.893)		3.636 (0.477–27.733)		3.207 (0.665–15.469)		2.682 (0.534–13.487)	
N stage		0.429		0.284		0.068		0.022
N1	1.000		1.000		1.000		1.000	
N2	1.327 (0.658–2.674)		1.504 (0.712–3.176)		2.144 (0.946–4.861)		3.358 (1.194–9.446)	
Adjuvant chemotherapy		<0.001		0.010		0.029		0.011
No	1.000		1.000		1.000		1.000	
Yes	0.254 (0.122–0.529)		0.341 (0.151–0.774)		0.371 (0.152–0.904)		0.197 (0.057–0.687)	
No. of LNs dissected		0.218				0.676		
≥76	1.000				1.000			
<12	1.681 (0.736–3.841)				1.364 (0.318–5.846)			
Pathological grading		0.550				0.747		
Well/moderate	1.000				1.000			
Poor/anaplastic	0.451 (0.107–1.909)				0.865 (0.360–2.082)			
Venous invasion		0.936				0.567		
Negative	1.000				1.000			
Positive	0.874 (0.420–1.822)				1.277 (0.553–2.952)			
Perineural invasion		0.772				0.872		
Negative	1.000				1.000			
Positive	1.114 (0.536–2.315)				0.930 (0.382–2.263)			
CEA (ng/ml)		0.487				0.034		0.135
≤1	1.000				1.000		1.000	
>5	1.264 (0.653–2.449)				1.411 (0.547–3.641)		1.433 (0.542–3.788)	
MMR status		0.294				0.557		
pMMR	1.000				1.000			
dMMR	0.043 (0.000–15.492)				1.210 (0.641–2.283)			
25(OH)D level (ng/ml)		0.020		0.043		0.024		0.012
Low	1.000		1.000		1.000		1.000	
High	0.430 (0.211–40.875)		0.474 (0.230–0.978)		0.289 (0.098–0.850)		0.233 (0.075–0.727)	

AJCC/UICC TNM staging system is highly related to T/N stage, there it is not included in multivariate analysis.

25(OH)D, 25-hydroxyvitamin D3; HR, hazard ratio; LN, lymph node; CEA, Carcinoembryonic antigen; pMMR, proficient Mismatch Repair; dMMR, different Mismatch Repair.

In the validation cohort, for left-sided CRC, serum 25(OH)D level was positively correlated with OS (p=0.024, **Figure 4B**). Univariate analysis showed that OS was associated with serum 25(OH)D level, T stage, adjuvant chemotherapy, and CEA level (p<0.05). These factors were included in the multivariate analysis, but only serum 25(OH)D level (HR 0.233, 95% CI 0.075–0.727, p=0.012), T stage (HR 2.991, 95% CI 1.219–7.337, p=0.007), N stage (HR 3.358, 95% CI 1.194–9.446, p=0.022) and adjuvant chemotherapy (HR 0.197, 95% CI 0.057–0.687, p=0.011) were identified as independent factors (**Figure 4B**, **Table 4**). For right-sided CRC, there was no significant association between serum 25(OH)D level and OS (p=0.08, **Table S2**).

## Discussion

In recent years, the relationship between 25(OH)D level and tumorigenesis has received increasing attention, especially in CRC. In pilot retrospective studies, higher 25(OH)D levels were associated with improved overall survival, as shown in all colorectal cancer patients (10–12). Vitamin D deficiency is very common in stage IV patients receiving first-line chemotherapy, as well as in black and female colorectal cancer patients (13).

However, vitamin D status has been less studied in Asian populations. Japanese studies on lung cancer indicated that vitamin D supplementation in NSCLC patients can improve the survival of patients with early-stage lung adenocarcinoma and a 25(OH)D lower level (20), and another study revealed that higher preoperative 25OHD levels may be associated with a better survival rate for patients with colorectal cancer. Few studies have focused on the effect of vitamin D status on the survival of patients with stage II and III colon cancer and recurrence, especially in the Asian population.

However, epidemiological studies have revealed that the overall vitamin D level in the Asian population is low, showing a high proportion of deficiency and insufficiency. As found in the present study, 58.5% of the study population lacked vitamin D (<50 nmol/L), 30.4% had a vitamin D deficiency (<75 nmol/L), and only 11.1% had levels in the normal range (≥75 nmol/L). The cut-off point of 50 nmol/L in previous European and American studies 10,13,18–20 was not completely applicable to the Asian population. The research participants of those studies were mostly between 60 and 70 years old (8, 10, 13, 21, 22, 23), which is within the same range for the studied cohort of this study (61 years old, range, 20–90, IQR, 52, 68; range, 28–88, IQR, 52, 67). However, the mean BMI of the studies European and American populations (range, 26.1–29.9) was much higher than that of the cohort of this study (22.7, range, 16.02–26.6, IQR, 20.3, 25.0). This is also consistent with conventional nutritional data and knowledge of European and American population groups and Asian population groups. Therefore, this study used the median level to define the serum 25(OH)D cut-off for the Asian population, thus excluding the errors caused by selecting the maximum P value as the cut-off point.

Serum was extracted from the latest blood samples collected from patients before surgery, which excluded reverse causality caused by patient status. However, the test data showed that the serum 25(OH)D level of the studied population was indeed lower than that of the normal population. After excluding the impact of surgery, treatment, a possible explanation for this vitamin D deficiency is that the included patients had low BMIs and were generally older [61 (range, 20–90, IQR, 52, 68; range, 28–88, IQR, 52, 67), 56.4% were over 60 years old], resulting in less overall exposure to sunlight, participation in fewer outdoor sports, weaker body metabolism and increased calcium loss. Women accounted for 41.9% of this cohort, and vitamin D levels are generally lower in postmenopausal women. This could possibly reflect that the decrease in vitamin D levels is causally related to the diagnosis of CRC.

In addition, vitamin D involved in other pathway contributing to progress of CRC, including 1α,25-dihydroxy-vitamin D(3) [1α,25(OH)(2)D(3)] inhibition of mitogen-activated protein kinase (MAPK)-extracellular signal-regulated kinase (ERK) signaling through the suppression of epidermal growth factor (EGFR) and insulin-like growth factor 1 (IGF1), which induces apoptosis through the IGFR1-phosphatidylinositol 3-kinase (PI3K)-Akt-dependent signaling pathway (24).

The results showed that vitamin D status was positively correlated with OS in patients with left-sided stage III CRC but not in those with right-sided disease, which may be related to the participation of vitamin D in the MAPK pathway. Right-sided CRC often shows MAPK pathway activation. When the 25(OH)D level is lower than the median value, although its active product 1α,25(OH)(2)D(3) loses its inhibitory effect on MAPK-ERK signaling, MAPK itself is mostly activated. Moreover, according to RAS activating mutations may be less responsive to vitamin D mediated treatment or chemoprevention (25). The weakened inhibitory effect has a subtle impact on the prognosis of right-sided CRC. In contrast, left-sided CRC has a relatively weaker activation of the MAPK pathway, and the active product 1α,25(OH)(2)D(3) of 25(OH)D suppresses MAPK-ERK signaling, impacting tumor differentiation, apoptosis and growth.

Although this study has made new progress, it still has some limitations. First, the research design is retrospective. There may be certain amount of degradation of 25(OH)D in the serum during long-term frozen storage and rethawing, but this degradation effect will be almost the same if the serum is preserved and rethawed under the same conditions. Second, our research was conducted with a single-center cohort. Although internal cohort verification was performed to prevent overinterpretation, verification with multiple cohorts is a better choice to confirm whether this research result is universal. In addition, although CRC was stratified into stages II and III with a variety of influencing factors, the sample size was comparatively small. Therefore, the power still needs to be verified.

In conclusion, the tested data estimated the distribution and cut-off vitamin D levels in Asian patients with stage II and III colorectal cancer. Several analyses indicated that vitamin D status is positively correlated with the survival of CRC patients, especially left-sided stage III CRC patients. Additional efforts are required to understand the underlying mechanisms and optimal therapy methods.

## Data Availability

The original contributions presented in the study are included in the article/**Supplementary Material**. Further inquiries can be directed to the corresponding author.

## Ethics Statement

This study was approved by the Institutional Review Board of FUSCC. All patients provided written informed consent.

## Author Contributions

YB contributed to the study design, data analysis, statistical analysis, manuscript preparation, and editing. YL contributed to the study design, data analysis, and funding acquisition. YG acquired data and contributed to the quality control of the data and algorithms. QH prepared the manuscript. SC reviewed the manuscript. JP contributed to the study concepts, manuscript review, and funding acquisition. All authors contributed to the article and approved the submitted version.

## Funding

This research was funded by the Shanghai Sailing Program(19YF1409500 to YL), Shanghai Anticancer Association EYAS PROJECT (SACA-CY1A05 to YL), Science and Technology Commission of Shanghai Municipality (18401933402 to JP), National Natural Science Foundation of China (Grants No. 81672374 to Cai), National Natural Science Foundation of China (Grants No. 82002946 to YL) and National Natural Science Foundation of China (Grants No. U1932145 to JP).

## Conflict of Interest

The authors declare that the research was conducted in the absence of any commercial or financial relationships that could be construed as a potential conflict of interest.

## Supplementary Material

Supplementary Figure 1 Seasonal analysis of serum 25(OH)D levels in primary and validation cohorts.

Supplementary Figure 2 Proportional hazards assumption is stated in both primary and validation cohorts for valid estimates from Cox proportional hazards models.
