# Supplementary figures and images for: Vitamin D Status and Survival in Stage II-III Colorectal Cancer
Source: Front Oncol. 2020 Dec 17;10:581597. doi: 10.3389/fonc.2020.581597 (PMC7773833; doi:10.3389/fonc.2020.581597)

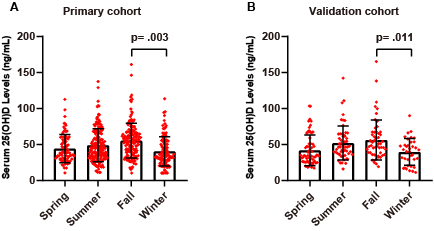

Supplement: Supplementary Figure 1 — Seasonal analysis of serum 25(OH)D levels in primary and validation cohorts. [file Image_1.tif]

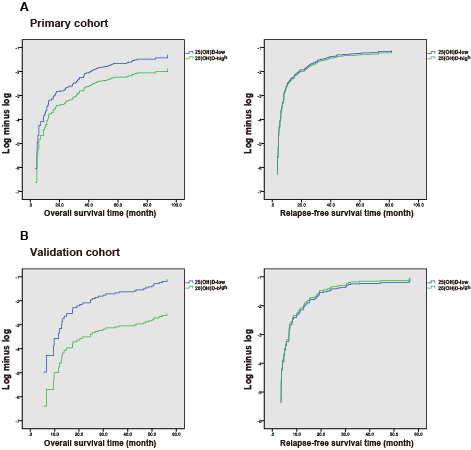

Supplement: Supplementary Figure 2 — Proportional hazards assumption is stated in both primary and validation cohorts for valid estimates from Cox proportional hazards models. [file Image_2.tif]
