# Supplementary material for: Vitamin D Status and Survival in Stage II-III Colorectal Cancer
Source: Front Oncol. 2020 Dec 17;10:581597. doi: 10.3389/fonc.2020.581597 (PMC7773833; doi:10.3389/fonc.2020.581597)
Supplement: Supplementary file 3 [file Table_1.docx]

**TableS1.** Multivariate Cox Regression Analysis for Overall Survival of CRC in Stage II Disease

|  | Primary | |
| --- | --- | --- |
| Characteristic | Univariate analysis | P |
|  | HR (95%CI) |  |
| Age |  | .044 |
| <60 | 1.000 |  |
| ≥60 | 3.030 (1.030-8.910) |  |
| Sex |  | .698 |
| Male | 1.000 |  |
| Female | 0.844 (0.358-1.990) |  |
| Histology |  | .593 |
| Adenocarcinoma | 1.000 |  |
| Mucinous tumors | 1.311 (0.487-3.531) |  |
| Primary site |  | .978 |
| Right-sided | 1.000 |  |
| Left-sided | 1.012 (0.444-2.308) |  |
| T stage |  | .291 |
| T3 | 1.000 |  |
| T4 | 1.572 (0.679-3.640) |  |
| Adjuvant chemotherapy |  | .128 |
| No | 1.000 |  |
| Yes | 0.527 (0.231-1.203) |  |
| No. of LNs dissected |  | .127 |
| ≥12 | 1.000 |  |
| <12 | 2.571 (0.764-8.660) |  |
| Pathological grading |  | .889 |
| Well/moderate | 1.000 |  |
| Poor/anaplastic | 0.962 (0.326-2.844) |  |
| Venous invasion |  | .802 |
| Negative | 1.000 |  |
| Positive | 0.507 (0.068-3.766) |  |
| Perineural invasion |  | .984 |
| Negative | 1.000 |  |
| Positive | 0.896 (0.266-3.014) |  |
| CEA (ng/ml) |  | .590 |
| ≤5 | 1.000 |  |
| >5 | 1.541 (0.675-3.515) |  |
| MMR status |  | .940 |
| pMMR | 1.000 |  |
| dMMR | 1.212 (0.412-3.564) |  |
| 25(OH)D level (ng/ml) |  | .358 |
| Low | 1.000 |  |
| High | 0.659 (0.271-1.603) |  |

NOTE.

AJCC/UICC TNM staging system is highly related to T/N stage, there it is not included in multivariate analysis.

Abbreviations: 25(OH)D, 25-hydroxyvitamin D3; HR, hazard ratio; LN: lymph node; CEA: Carcinoembryonic antigen; pMMR: proficient Mismatch Repair; dMMR: different Mismatch Repair.
