# Supplementary material for: Vitamin D Status and Survival in Stage II-III Colorectal Cancer
Source: Front Oncol. 2020 Dec 17;10:581597. doi: 10.3389/fonc.2020.581597 (PMC7773833; doi:10.3389/fonc.2020.581597)
Supplement: Supplementary file 4 [file Table_2.docx]

**TableS2**. Univariate and Multivariate Cox Regression Analysis for Overall Survival of CRC in Stage III Right-Sided Disease.

|  | Primary | | Validation | |
| --- | --- | --- | --- | --- |
| Characteristic | Univariate analysis | P | Univariate analysis | P |
|  | HR (95%CI) |  | HR (95%CI) |  |
| Age |  | .370 |  | .647 |
| <60 | 1.000 |  | 1.000 |  |
| ≥60 | 1.488 (0.624-3.548) |  | 0.781 (0.271-2.250) |  |
| Sex |  | .693 |  | .464 |
| Male | 1.000 |  | 1.000 |  |
| Female | 1.187 (0.507-2.781) |  | 0.676 (0.237-1.929) |  |
| Histology |  | .050 |  | .091 |
| Adenocarcinoma | 1.000 |  | 1.000 |  |
| Mucinous tumors | 2.319 (1.001-5.371) |  | 2.570 (0.860-7.682) |  |
| T stage |  | .282 |  | .001 |
| Tis-T2 | 1.000 |  |  |  |
| T3 | 0.183 (0.016-2.026) |  | 1.000 |  |
| T4 | 0.923 (0.215-3.972) |  | 6.483 (2.209-19.024) |  |
| N stage |  | .009 |  | .002 |
| N1 | 1.000 |  | 1.000 |  |
| N2 | 3.123 (1.333-7.315) |  | 5.511 (1.841-16.491) |  |
| Adjuvant chemotherapy |  | .949 |  | .507 |
| No | 1.000 |  | 1.000 |  |
| Yes | 0.961 (0.284-3.252) |  | 0.690 (0.231-2.063) |  |
| No. of LNs dissected |  | <.001 |  | .283 |
| ≥12 | 1.000 |  | 1.000 |  |
| <12 | 13.097 (3.349-51.209) |  | 3.063 (0.396-23.678) |  |
| Pathological grading |  | .311 |  | .399 |
| Well/moderate | 1.000 |  | 1.000 |  |
| Poor/anaplastic | 1.973 (0.816-4.772) |  | 1.359 (0.444-4.159) |  |
| Venous invasion |  | .610 |  | .012 |
| Negative | 1.000 |  | 1.000 |  |
| Positive | 1.248 (0.533-2.923) |  | 5.136 (1.426-18.496) |  |
| Perineural invasion |  | .041 |  | .502 |
| Negative | 1.000 |  | 1.000 |  |
| Positive | 0.112 (0.014-0.918) |  | 0.645 (0.180-2.317) |  |
| CEA (ng/ml) |  | 1.000 |  | .780 |
| ≤5 | 1.000 |  | 1.000 |  |
| >5 | 1.001 (0.434-2.310) |  | 0.898 (0.422-1.912) |  |
| MMR status |  | .333 |  | .286 |
| pMMR | 1.000 |  | 1.000 |  |
| dMMR | 0.371 (0.050-2.762) |  | 0.348 (0.045-2.695) |  |
| 25(OH)D level (ng/ml) |  | .396 |  | .097 |
| Low | 1.000 |  | 1.000 |  |
| High | 0.677 (0.276-1.664) |  | 0.179 (0.023-1.366) |  |

NOTE.

AJCC/UICC TNM staging system is highly related to T/N stage, there it is not included in multivariate analysis.

Abbreviations: 25(OH)D, 25-hydroxyvitamin D3; HR, hazard ratio; LN: lymph node; CEA: Carcinoembryonic antigen; pMMR: proficient Mismatch Repair; dMMR: different Mismatch Repair.
